# Perceptions and Experiences about Self-Disclosure of HIV Status among Adolescents with Perinatal Acquired HIV in Poor-Resourced Communities in South Africa

**DOI:** 10.1155/2016/2607249

**Published:** 2016-09-08

**Authors:** Sphiwe Madiba, Mathildah Mokgatle

**Affiliations:** ^1^School of Public Health, Department of Environmental and Occupational Heath, Sefako Makgatho Health Sciences University, Pretoria, South Africa; ^2^School of Public Health, Department of Biostatistics, Sefako Makgatho Health Sciences University, Pretoria, South Africa

## Abstract

*Background.* There is limited research on the disclosure experiences of adolescents with perinatal acquired HIV (PAH). The study explores how adolescents with PAH experience living with HIV and examined their perceptions and experiences regarding disclosure and onward self-disclosure to friends and sexual partners.* Methods.* Thematic analysis was used to analyze in-depth interviews conducted with 37 adolescents.* Findings.* Adolescents received disclosure about their status at mean age of 12 years. They perceived disclosure as necessary and appreciated the truthful communication they received. Adolescents have learned to accept and live with HIV, and they desired to be healthy and normal like other people. After receiving disclosure, they found their treatment meaningful, and they adhered to medication. However, they also expressed a strong message that their HIV status was truly their secret and that self-disclosure to others will take the feeling of being normal away from them because they will be treated differently.* Conclusion.* Adolescents maintained secrecy in order to be accepted by their peers but also to protect themselves from stigma and isolation. Given that adolescents want to be informed of their HIV status but desire controlling self-disclosure of their HIV status, these should form the basis for development of disclosure interventions.

## 1. Introduction

The increasing availability and provision of free antiretroviral treatment (ART) have resulted in a significant reduction in mortality of HIV-infected children, surviving through childhood into adolescence in low income countries [1, 2]. The increased survival of children and adolescents with perinatally acquired HIV (PAH) has been accompanied by unique needs and specialized management [3]. Their needs are more sensitive and varied than those of adults; they must simultaneously deal with adult issues, such as disclosure, stigma, and practicing safe sex [4]. However, adolescents with PAH can deal with all of these issues only if they are informed about their HIV diagnosis. Yet, the World Health Organization (WHO) maintains that most of the estimated 2 million adolescents with PAH worldwide are unaware of their HIV status [5]. The levels of disclosure of the HIV status to adolescents are low even though limited research shows that adolescents prefer to be informed about their HIV status [6–8].

While adolescents and children with PAH prefer to be informed about their HIV diagnosis, data from studies that examine self-disclosure by adolescents suggest that disclosure to sexual partners and friends remains low, particularly in developing countries [9–11]. Self-disclosure to sexual partners is considered an important process in preventing HIV transmission. It is for these reasons that the disclosure focus is shifting from caregivers' disclosure to HIV-infected children to decisions concerning self-disclosure among adolescents with PAH. The importance of self-disclosure increases as children with PAH progress into adolescence, and concerns regarding HIV transmission intensify [12]. Self-disclosure among adolescents with PAH increases condom negotiation and use, improves ART adherence, and reduces levels of unprotected sexual activities [13]. Thoth and colleagues argued that disclosure to sexual partners is perhaps the most important form of disclosure from a public health standpoint [14]. Studies that have explored the link between self-disclosure and adherence showed that disclosure gives meaning to adherence [6, 15]. Therefore, the reluctance of adolescents with PAH to disclose their status to their sexual partners raises concerns about sexual transmission of HIV and could undermine HIV prevention efforts [16].

Research has shown that self-disclosure among adolescents with PAH is a complex and difficult decision [12, 17]. They are often reluctant to disclose their status for fear of rejection, stigmatization, negative reactions, and responses [9, 12, 16, 17]. As a consequence, they put a lot of effort into maintaining the secrecy of their status and want to have control regarding to whom they disclose their status [12]. Determining who should know about their status and choosing when and how to disclose their status to them may help adolescents feel more empowered and autonomous [16]. For adolescents who defer onward self-disclosure, keeping silent is perceived as a behavior that helps to protect themselves [18].

Self-disclosure among adolescents with PAH is also constrained by parental negative attitudes toward disclosure, which might include directives that the adolescent should keep the status secret [12]. This suggests that children and adolescents with PAH lack full autonomy over the decision for self-disclosure because onward disclosure simultaneously exposes the serostatus of their biological mother. As a result, adolescents with PAH whose biological parents hold negative attitudes toward disclosure are less likely to disclose their status to others, including sexual partners [12, 17, 19, 20].

There are limited available data reporting the disclosure experiences of children and adolescents with PAH from the perspectives of their caregivers. While it is important for adolescents to disclose their status to significant others, little has been published on their perspectives and experiences of knowing their HIV positive status [7, 8]. The study explores how adolescents with PAH experience living as HIV positive adolescents and examined their perceptions and experiences about disclosure and onward self-disclosure to friends, sexual partners, and others. Understanding the process of disclosure and its consequences for adolescents is critical for determining what types of support adolescents and parents need in order to disclose their status [16].

## 2. Methods

### 2.1. Study Design

This was a qualitative exploratory design, using in-depth interviews with children and adolescents on ART who were aware of their HIV status. The study was conducted from December 2012 to July 2013 in health facilities in Mpumalanga and Gauteng provinces, South Africa. Data were collected in 3 ART clinics which were based in 3 community hospitals and 7 primary healthcare (PHC) clinics providing ART services to adults and children living with HIV. The study was conducted during the period when children and adolescents with PAH could be given ART in PHC facilities through the nurse-initiated and managed antiretroviral treatment (NIMART) initiative. Nurses in PHC facilities were trained to initiate ART, and the hospitals initiated the referral of children and adolescents on ART to PHC facilities. The health facilities were located in 3 subdistricts: one urban and 2 rural. The majority of the health facilities, including the 2 community hospitals, were located in the rural subdistricts of Nkangala district in Mpumalanga province, whereas the urban facilities were located in Tshwane district, Gauteng province.

### 2.2. Data Collection

A purposive sample of children and adolescents aged 12 to 18 years who acquired HIV perinatally were invited to take part in the study when they attended their medical appointments. Adolescents were selected consecutively until data saturation was reached. To ensure that the adolescents were fully aware of their status, comprehensive information about the purpose of the study was provided to the caregivers and healthcare workers (HCWs) and permission to interview the child or adolescent was obtained from the caregivers after the interviewers had confirmed that the child or adolescent knew their HIV status to prevent accidental disclosure. For this reason, children and adolescents who were accompanied by caregivers for their medical appointments were predominantly recruited and interviewed. The caregivers provided consent for children and adolescents who were below 18 years old, and they signed an assent form. Adolescents aged 18 years signed their own consent; these adolescents were often not accompanied by their caregivers, but the HCWs were aware of their disclosure status and pointed out those who were aware of their HIV status to the interviewers. In-depth interviews focused on the children's and adolescents' experiences of disclosure, everyday personal experiences of living as a person with HIV, and self-disclosure to friends and sexual partners for those who were involved in romantic relationships. The adolescents were provided comprehensive information about the study objectives, confidentiality of the interview, and voluntary participation prior to conducting the in-depth interviews. Individual in-depth interviews were conducted by the investigators and research assistants experienced in qualitative data collection. The interviews were conducted in 2 local languages (Setswana and IsiZulu), using a semistructured interview schedule that allowed the interviewers to be consistent but also flexible in the use of probing and follow-up questions. All individual interviews were conducted in the health facilities in private, were audio-recorded, and lasted about 30 minutes. At the end of the interviews, demographic information regarding each participant's age, gender, school grade, and duration on ART, type of caregiver, and adherence to medication was collected.

### 2.3. Data Analysis

The interviews were digitally recorded and then transcribed verbatim and translated to English for thematic analysis. The investigators (Sphiwe Madiba and Mathildah Mokgatle) independently read a number of transcripts repeatedly to identify the codes occurring most frequently across transcripts. The investigators met several times to reconcile emerging codes and develop a codebook. Using the initial codebook, the investigators coded a few transcripts together to refine the codebook. Once consensus was reached on the definitions of the themes in the codebook, the transcripts were imported into NVivo version 10, a qualitative analysis software package for the application of codes. Analysis continued with multiple readings of interviews to identify new themes and refine those in the codebook. Data was subsequently grouped under themes and subthemes for interpretation.

To ensure trustworthiness, we conducted the in-depth interviews in the local language and transcribed the interviews verbatim. Each transcribed interview was reviewed for accuracy by replaying each interview tape while reading and translating the transcripts. Coding by both the investigators reduced the potential bias that is associated with singular analysis. NVivo 10 computer software for qualitative data analysis was used to aid data organization and analysis [21].

### 2.4. Ethical Consideration

Ethical approval was granted by the University of Limpopo Research and Ethics Committee, Medunsa Campus. Permission was obtained from the Tshwane district, Mpumalanga province, and the Nkangala district, as well as the chief executive officers of the community hospitals and the managers of the PHC facilities. Informed consent was obtained from the adolescents and their caregivers; adolescents provided written informed consent or assent if they were below 18 years of age, and caregivers provided written informed consent for adolescents below 18 years of age. Privacy and confidentiality were safeguarded, and participation was voluntary.

## 3. Findings

### 3.1. Description of Study Participants

Thirty-seven adolescents participated in the study; 14 were under 15 years of age and 23 were aged 15 to 18 years. The mean age of the adolescents was 15.5 years, and the range was 12 to 18 years. There were more females (22) than males (15). Twelve out of 37 adolescents lived with their mothers alone, 6 with both parents, 3 with their fathers alone, 9 with their grandmothers, and 7 with older siblings and aunts. Fifteen adolescents were maternal orphans. Thirty-five adolescents were attending school; 11 were enrolled in primary school, while 24 were in secondary school (Table 1).

All the adolescents were formally informed about their HIV diagnosis at home and at the clinic or hospital, only one adolescent was not formally informed about his status; he overheard the nurse telling his mother at the clinic. Formal disclosure occurred at a variety of ages ranging from 5 to 16 years of age. Most (25) adolescents had known about their diagnosis for more than 5 years, and the mean duration since disclosure was 3.83 years (SD = 2.71, range of 1 to 10 years). Disclosure for most of the adolescents was preceded by episodes of serious illness; adolescents reported that they were sick or admitted to hospital when they were informed about their HIV status. With regard to onward self-disclosure, only 8 out of 37 adolescents had disclosed their HIV status to other people; four disclosed it to a friend or friends and four disclosed it to a teacher; none of the five adolescents who indicated that they had romantic partners during the interview had disclosed their status to the romantic partners (Table 2).

All the adolescents were on ART at the time of data collection for an average of 4.6 years (SD = 2.94, range of 1 to 10); none were on ART since birth. Most (21 out of 37) adolescents began ART shortly after disclosure. Seven reported that they missed an ART dose in the current month (Table 2).

### 3.2. Themes

Ten key themes contributed to the experience of the adolescents: perceptions of disclosure, reaction to disclosure, yearning for answers, finding justification for disclosure, having to take lifelong treatment, desire for a healthy life, learning to get on with life, being constantly reminded of HIV, keeping the secret, and self-disclosure. These are outlined below.

#### 3.2.1. Perceptions of Disclosure

Responding to whether children and adolescents should be told about their HIV positive status, adolescents felt that disclosure to children was a good thing to do.

*I think it's important [to disclose] because everybody must know about their status, and for me, knowing my status I see it as an important thing. (Male participant; 13 years old)*

*Children must be told about their HIV status, because if it is transmitted to children, you may find out that children do not know that they have HIV, and after that they get sick. (Female participant; 12 years)*

*It is better that they did not hide it from me…, they told me straight that I am HIV positive so that I don't stop taking treatment. (Female participant; 13 years old)*

Some of the adolescents also felt that disclosure to other people in general was important.

*I think not telling people [about HIV status] is not a right thing because people will never be educated about HIV. (Female participant, 15 years old)*

* I think it is better to tell people about your status so that they know, some people might judge you, but most of them will support you. I don't think it's a bad thing actually to tell your friend or your best friend because if really she or he is your friend then they will support you. (Male participant, 18 years old)*

On the other hand, some of the adolescents felt that disclosure to other people is not such a good idea.

*You must only tell your family members who will not tell other people outside because others are gossipers and will tell other people that this child is HIV positive. (Female participant, 14 years old)*

*It must be a secret. It is not something that you can just tell everyone. People outside my immediate family do not know. (Male participant, 18 years old)*

#### 3.2.2. Reaction to Disclosure

Adolescents experienced both positive and negative effects of disclosure. Some of them commented that the disclosure event was extremely shocking and hurtful, and they cried for days after learning that they had HIV. Some reacted with anger, pain, confusion, and disbelief and felt like they were dying at the time of the disclosure.

*I cried… I was very hurt, I was heartbroken. I was hurting and crying for five days. My aunt was trying to comfort me, but it was too painful. She kept talking to me. She was aware that I was hurting. (Female participant; 14 years old)*

*I was angry… crying…. I wanted to know why they did not tell me all this time, saying that I am still a child and I won't understand. (Male participant; 18 years old)*

*I felt like there is no one who loves me. It's like the universe has turned on me and then I felt angry deep inside my heart. (Female participant; 13 years old)*

*When I discovered about my HIV status, I did not feel good at all. You think about a lot of things. You think that you no longer have a life. You feel that people can see that you are HIV positive. (Male participant; 17 years old)*

Some of the adolescents were satisfied with how their parents or caregivers handled the disclosure and reported positive reactions, such as relief and happiness at learning the cause of their illness.

*I was happy that she told me so that I can know that I was suffering from HIV. She did not hide it from me. (Female participant; 14 years old)*

*What I liked is that she told me straight. She did not beat around the bush, and she told me straight, I understood after she told me. (Male participant; 18 years old)*

A few participants also reported a neutral reaction; that is, they felt nothing after learning their HIV status.

*Actually, I did not understand anything, I didn't understand what HIV positive was…. I was just taking the pills that they gave me. It never affected me. I just accepted. I just accepted…. I didn't have problems. (Female participant; 13 years old)*

*At that time, I was still very young. I think I was about 6 or 7 years old. I did not feel any pain or sorrow because I did not know anything. (Female participant; 18 years old)*

#### 3.2.3. Yearning for Answers

As already stated, discovering that they were HIV positive was shocking for some of the adolescents who could not understand what was happening to them. Some started questioning why them and how they were infected. What was confusing for the adolescents was that they never thought that they could be infected with HIV and could not figure out how it happened.

*I tested at the clinic, and they told me that I am HIV positive. I did not believe them, and I asked how I got it because I am still very young, and I have not slept with any man. (Female participant; 14 years old)*

*I was very shattered, and I asked myself what I did before God that I should be infected with HIV. (Male participant; 17 years old)*

*The thing is… I was asking myself how it came about, I could not figure out how I got this illness. (Male participant; 17 years old)*

*I ask myself why me? I ask myself questions that I could not answer… why me? I keep asking myself these questions. (Male participant; 15 years old)*

#### 3.2.4. Finding Justification for Disclosure

The adolescents appreciated the open and truthful communication they received during disclosure. Although most reacted to the disclosure with shock, confusion, pain, anger, and disbelief, they later found good reasons why disclosure occurred. Generally, one of the main reasons given to justify why children should receive disclosure was adhering to medication once treatment begins. Adolescents commented that disclosure was necessary to ensure that they stay alive.

*I think it [disclosure] was good because if ever they did not tell me, I would not be here today. I saw it as being important for them to tell me, so that I can know my status. (Female participant; 13 years old)*

*I am saying that knowing is better…. If I did not know, I would not be alive right now… because I had told myself that I was dying. (Male participant; 18 years old)*

*What I am happy about is… if I did not know that I am HIV positive, I would probably be thin or have died. (Female participant; 15 years old)*

#### 3.2.5. Having to Take Lifelong Treatment

After they learned their diagnosis, most adolescents commented that they have accepted that they will have to be on medication for the rest of their lives. They also reported that they learned to take their medications correctly to stay healthy. However, some of the adolescents acknowledged that they have no choice but to take their medications for the rest of their lives, as they were an essential part of their lives and felt that the medication makes them feel better.

*What I love about ARVs is that they save lives, our lives, and again what I love is that I am going to live more years than the ones that are expected. (Male participant; 17 years old)*

*They treat me good, okay, because after I started taking them, I no longer became sick. I can spend time with my friends and still be okay. (Male participant; 14 years)*

*I know when I take ARVs, there's no way I'm going back… There is no way that I cannot adhere to doctors' orders. I am doing what they told me and that which I know is good for my life. The good thing is that they add more days to your life, there's nothing that is not good, you are supposed to take your ARVs on time and you are not supposed to skip no matter what happens. (Female participant; 13 years old)*

While most of the adolescents felt that lifelong medication saves life, some felt differently and dreaded having to take medication for the rest of their lives.

*I was very angry. My heart was very sore that I was HIV positive and that I am supposed to take medication. I did not know that I was supposed to drink the pills, and it is for life. That is what hurt me the most. (Male participant; 17 years)*

*What is bad… the bad thing is that every time you have to drink pills, the pills reminds you about your illness. (Male participant; 15 years old)*

*I don't like taking ARVs…. I know they are very important for me, but sometime I tell my mother that I don't want to take them, but I end up drinking them. (Female participant; 18 years old)*

#### 3.2.6. Desire for a Healthy Life

The adolescents desired to be healthy and normal like other people. They commented that they take their medication correctly because they do not want to be sick. They conveyed a sense of positivity in their lives and that HIV does not have to be associated with ill health. Most understood the importance of taking care of their health.

*I am taking them [ARVs] correctly. I am taking them with understanding that I don't want to get sick. I don't want to stay at the hospital, so I take my pills accordingly. (Female participant; 15 years old)*

*There's nothing that I liked [about disclosure] because I did not want to have HIV/AIDS. I wish to be just like other people. I wish I did not have HIV, avoid going to the clinics throughout. (Female participant; 15 years old)*

*I feel okay now because I have to drink my pills correctly and if I take them correctly, I will live and also if I look after myself, I will be fine. (Male participant; 17 years old)*

#### 3.2.7. Learning to Get on with Their Lives

Adolescents conveyed a sense that they learned to live with their diagnosis and that they are getting on with their lives. They commented that HIV was something that they have learned to accept and live with and do not think about very often. They also expressed that they lead a normal life and that they were similar to other adolescents.

*I am just like everyone; there's nothing I can do. I live like a normal person. I don't know how HIV-positive persons live because at the moment I live like a normal person. (Male participant; 15 years old)*

*I come to their treatment, and I do everything they say I must do. I feel okay, and I don't have stress. I have even forgotten that I am ill. I have removed the knowledge that I am HIV positive out of my mind. (Female participants; 14 years old)*

*I feel as a normal person. I do things as a normal person, and I am taking my medications. (Male participant; 15 years old)*

#### 3.2.8. Being Constantly Reminded of HIV

Though most adolescents desired to live like normal people and have learned to accept that they have HIV, some commented that they were frequently reminded that they are living with HIV. For some, taking ART medication is a daily reminder of their condition.

*I don't want to keep that, I must stay without illnesses, but there are days when I can't help it, especially when I have to take my pills at 7 o'clock. (Female participant; 18 years old)*

*Even now, I still ask myself when I'm sick… I ask myself about the pills when the time comes to take them, I ask why…. (Female participant; 15 years old)*

*I am no longer feeling HIV positive because sometimes during the day you find that I forget that I have that thing [HIV]. I would have taken my pills and be okay. I feel fine then I remember that I am sick. (Female participant; 18 years old)*

One adolescent narrated how he wanted to remember that he is HIV positive but also celebrate that he is alive, as he reflected the following:

*I have told myself that every single year on my birthday I celebrate…. This year I am 18 years old so I am going to say I have been living with HIV for 18 years. I am celebrating that at least the years are passing. (Male participant; 18 years old)*

#### 3.2.9. Keeping the Secret

Adolescents were aware of the secret nature of their HIV diagnosis. They reported that they consider their HIV diagnosis a secret and do not feel a need to disclose their status. They further commented that HIV is an issue that they do not commonly talk about with friends, and most decided to keep it secret. Fear of being gossiped about and stigmatized was an important consideration for keeping their HIV status secret.

*It's my own secret. I am not supposed to tell my friends. (Male participant; 16 years old)*

*My status is my secret and will remain my secret until I am older. In most cases, it is a secret; the only people who need to know are your family. (Male participation; 17 years)*

*I asked myself that if I'm going to tell them about my status, what's going to happen? So I decided to keep quiet. (Male participant; 16 years old)*

Some of the adolescents were given instructions not to talk:

*They said [my father] I must not tell them [friends] because they are going to tell people.*

*My grandmother told me that it's a secret, and I must not tell anyone. (Female participant; 17 years old)*

Disclosure to friends resulted in stigmatization for some of the adolescents who felt the need to disclose their status to friends. The subsequent experience of stigma led to secrecy and emotional trauma for the adolescents.

*I did not know whom to tell, my mother was always crying, always under stress, and I did not know whom to tell. I told myself that I have to break the silence once and for all, then I told my friends, and they started isolating me. (Female participation; 14 years)*

*I told my friend, and after I told him, he told others; whenever I pass them they bothered me. (Male participation; 15 years)*

However, some of the adolescents who disclosed their status to friends were supported:

*I have good friends, and they know about my status. They support me, and sometimes I forget that I am even HIV positive and things like that. (Female participant; 16 years old)*

*Me, I tell them, I don't have a secret. I want them to know, but only my best friends. They need to know so they can support me and even to remind me to take my pills. They should remind me. However, ordinary people do not know yet. (Female participant; 14 years old)*

#### 3.2.10. Self-Disclosure

When asked whether adolescents had told their romantic partners and friends at school about having HIV, they commented that there are many things they talk about, but they usually do not talk about HIV. They further felt that it was not important to tell people about their HIV status because their status is their secret. They were certain that they could not tell their friends about their HIV status and expressed anxiety at how difficult disclosure would be.

*What I do not like about knowing that I have HIV is having to tell other people that I have HIV; it's not important to tell them. (Female participant; 15 years old)*

*There are many things you can talk about with friends, but HIV… no…. (Male participant; 14 years old)*

*If you have a heart, you can tell them. I have not yet told anyone. (Female participant; 17 years old)*

*It will be too painful to me to tell my friends because they will perceive me differently. (Female participant; 12 years old)*

Although they were uncertain of the reactions of others to the disclosure, adolescents expressed fear that disclosure to romantic partners and friends would lead to rejection and isolation. Their comments suggested that they feared that their romantic partners and friends would react in a negative way.

*If I tell him, he will leave me. (Female participant; 18 years old)*

*I do want to tell him, but eish… I don't think he will understand. (Female participant; 18 years old)*

*I think if I tell my friends that I have HIV, they won't want to play with me any longer, and they will laugh at me. (Female participation; 15 years old)*

*I don't want them judging me, and I am afraid that my best friend will refuse to play with me. (Male participation; 14 years old)*

The nature, length, or quality of the relationship was an important consideration for disclosure as expressed by one of the adolescents who was engaged in a romantic relationship.

*I will wait and see how far our relationship goes. I am waiting for the right time, when the right time comes, I will tell him [my boyfriend]. (Female participant; 18 years old)*

Adolescents also expressed fear that disclosure would lead to people finding out about their HIV status accidentally without their permission. They did not trust that others would maintain the secrecy of their HIV status.

*I am scared that if I tell him [my boyfriend] he might tell other people. (Female participation; 18 years old)*

*I won't tell my friends because they are not trustworthy. I don't trust them, and they can go and tell everybody in this world, like at school. (Female participant; 13 years old)*

*I will not tell him (boyfriend)…, he has these friends, and you will find that sometimes after telling him, it will be all over the streets. (Female participant; 18 years old)*

## 4. Discussion

The study explored the experiences of adolescents with perinatally acquired HIV in low socioeconomic settings. We found that adolescents learned about their HIV diagnosis through the clinic or hospital and from their primary caregivers at home. More adolescents reported that they were informed about their HIV diagnosis by their primary caregivers in the home environment compared to healthcare providers in the hospital or clinic. Our findings are in contrast to other studies, which showed that most disclosure occurred in the healthcare environment [22, 23]. However, the disclosure practices in the current study are in line with the recommendation that primary caregivers take a leading role in disclosure, while healthcare providers provide additional information and ongoing counseling and support [24–27]. Despite being perinatally infected, most adolescents learned about their HIV diagnosis at varying ages with a mean age of 12 years. The mean age of disclosure in the current study is consistent with previous research [12]. Similar to previous studies, most adolescents were diagnosed after lengthy periods of illness, and disclosure was preceded by episodes of illness and hospitalization [22, 23, 28].

Most of the adolescents experienced negative emotions, such as shock, pain, anger, confusion, and disbelief following disclosure. They described the disclosure event as extremely shocking and hurtful, and some felt like they were dying. Although some reported that they cried for days after learning that they had HIV, these emotional reactions were brief. The short-lived emotional reactions observed are in line with previous findings from developing and developed countries, showing that disclosure does not seem to have major long-term negative psychological outcomes [16, 23, 28].

We also found that adolescents reported positive emotions, such as relief and happiness, after disclosure. They perceived disclosure positively because of the open and truthful communication through which disclosure was delivered. They were also appreciative of the disclosure because it helped them to understand their disease. These adolescents justified why disclosure had to happen; they conveyed a sense that disclosure saved their lives because they understood why they had to take their medication after disclosure. Our findings are consistent with what was reported in a study among adolescents in Tanzania and Botswana. The findings suggested that knowledge of HIV status provided HIV-infected adolescents with opportunities to take care of themselves and to adhere to treatment [6].

On the other hand, adolescents who experienced negative emotions after learning about their HIV status yearned for answers to understand how and why they were infected. They reported that they could not figure out how they could have been infected if they did not engage in sexual activities. They expressed ambivalent feelings of self-blame and wanted to know why them or what they did wrong to be punished with this disease. Similarly, adolescents in a study conducted in Zambia internalized blame after disclosure but thereafter adjusted positively [16]. This is in contrast to what was found among adolescents in Botswana and Tanzania, where the data revealed that the adolescents knew that they themselves were not to blame for their HIV status. The authors suggested that the lack of self-blame contributed to the adolescents' ability to handle HIV stigma [6].

After they learned their diagnosis, all adolescents reported that they understood why they had to take their medication correctly and accepted that they will have to be on medication for the rest of their lives to stay healthy. Similar to findings from other studies, receiving disclosure made adhering to ART meaningful, and adolescents reported that disclosure contributed significantly to their adherence to the ART [6, 22, 28]. We found that because adolescents desired to be healthy and normal like other people, they took their medication correctly to avoid becoming sick. Most of the adolescents understood the importance of taking care of their health. Midtbø and colleagues [6] also reported that, by adhering to treatment, the adolescents managed to take better control of their lives. In this study and others, the majority of adolescents began ART after prolonged illness and attributed the vast improvements in their health to the ART [29].

It should, however, be noted that a minority of adolescents in the current study acknowledged that they have no choice but to take their medications for the rest of their lives, as they were an essential part of their lives and make them feel better. This is consistent with recent data from Kenya, where HIV positive children and adolescents disliked taking their medications but appreciated their role in helping them stay healthy [28].

Similar to findings from a study conducted with young people in the United Kingdom [12] and Canada [30], adolescents have learned to accept and live with HIV. They conveyed a sense that they were similar to other adolescents. They felt that they lead a normal life. In addition, they were getting on with their lives, and HIV was something they did not think about every day. Adolescents in the Canadian study also depicted HIV as having a minimal effect on their everyday lives [30]. Our findings are also parallel to studies conducted in other developing settings, such as in Botswana and Tanzania, where disclosure gave the adolescents a better understanding of their life situation to continue to lead what they describe as a normal life [6, 22]. Midtbø and colleagues also maintained that after disclosure life made sense to the adolescents who found their treatment meaningful and wanted to adhere to the treatment to keep themselves healthy [6].

Although most adolescents in the current study conveyed a sense that HIV is something they do not think about every day, some commented that being on lifelong medication was a daily reminder that they were living with HIV. Despite the constant reminder, most of the adolescents desired to live like normal people. They felt that self-disclosure to friends and romantic partners would take the feeling of being normal away from them because they would be treated differently. Hogwood and colleagues argued that adolescents reject the notion of being different by maintaining secrecy of their HIV diagnosis, as HIV can be a hidden or invisible condition [12]. Similarly, most of the adolescents in the current study chose to keep their status a secret because peer acceptance was more important in their lives. According to Midtbø and colleagues [6], the majority of young people know the benefits of disclosing their status but do not want to risk rejection and isolation from peers. The fear of rejection and isolation emanates from the negative beliefs and stigma concerning HIV [12]. Parallel to other research, the main reason adolescents maintained secrecy was to protect themselves from stigma and isolation [16]. There is a need to modify public perception of disclosure to take into consideration the gains from maintaining secrecy for the adolescents, given how they feel about self-disclosure [31].

Although adolescents perceived being told about their HIV status positively, they had different views about onward self-disclosure to friend, romantic partners, and other people outside of their close family network. We found that adolescents expressed a strong message that their HIV status was truly their secret and they do not have to tell other people; as a result, only 8 out of 37 adolescents disclosed their status, and only 4 disclosed it to friends. This study and others show that adolescents are often reluctant to disclose their HIV status to their romantic partners and friends, mainly for fear of rejection and stigmatization [16, 22]. In line with previous studies, adolescents also expressed a desire to control when and to whom they disclose their HIV status as a way to prevent rejection, isolation, and stigma [16]. When adolescents know their HIV status, they are in a position to keep their status secret or decide to whom they choose to disclose their status. In this way, they are in a better position to deal and cope with stigma [6]. A Canadian study also showed that adolescents who know their HIV status were in control of to whom and when they disclose their HIV status. They believed that friends at school do not really need to know their HIV status [30].

### 4.1. Study Limitation

One potential limitation in this study is recall bias; the data were based on the children and adolescents responses, which might be subject to alterations in the perceptions of experiences because of the time effect between the disclosure event and data collection. We also recruited children who were accompanied to the clinic to prevent accidental disclosure to children who were not accompanied to the clinic by caregivers because we would not be able to verify that they knew their HIV status. These children might have different perceptions and experiences of disclosure. The adolescents reported that they were very sick when their status was disclosed, but we did not validate the information from the clinical records to be able to report the conditions of the adolescents at disclosure so we depended on self-reports from the adolescents.

The strength of the study is that it was carried out in multiple health facilities in rural and urban settings. Although the aim of a qualitative study is not to generalize, our data represent the experiences of adolescents and children in settings that could represent the general population of children with PAH receiving ART in public health facilities.

### 4.2. Conclusion

We found that, contrary to the belief by caregivers of children with PAH that disclosure will harm children and adolescents, most perceived disclosure positively and have learned to accept and live with HIV and felt that it has minimal effect on their lives, as they were similar to other adolescents. We also found that because adolescents desired to be healthy and normal like other people, after disclosure, they found their treatment meaningful, and they adhered to medication.

As most adolescents desired to live a normal healthy life, they perceived onward self-disclosure negatively and felt that they would be treated differently if they disclosed their HIV status to friends and romantic partners. Consequently, most maintained secrecy in order to be accepted by their peers. In their context, peer acceptance was much more important than disclosure of their HIV status, which most felt had nothing to do with their friends. Maintaining secrecy was also a way to protect themselves from stigma and isolation from others within the home and school environment.

In view of the negative perceptions of disclosure to others and the fact that adolescents desire to control onward self-disclosure of their HIV status, the development of disclosure guidelines for interventions to support adolescents to disclose their status should take these findings into consideration. Of concern is the potential transmission of HIV to sexual partners if perinatally infected adolescents do not have the skills and efficacy to negotiate and use condoms consistently when the time comes to engage in sexual activities. Continuous sexual risk reduction counseling should form part of their routine treatment and care so that they attain the skills to practice safe sex when they start engaging in sexual activities. It is also essential that HIV is taught as part of a broader curriculum, particularly to understand that HIV is a mother-to-child transmitted disease and not just a sexually transmitted disease. This will provide factual and accurate information to learners at school and reduce HIV stigma. Only when their peers understand perinatal transmission of HIV will perinatally infected children and adolescents find the school environment conducive for disclosure.

## Figures and Tables

**Table 1:** Demographic profile of adolescents and relationship with caregiver.

Variable	Number	Percent
*Gender*		
Female	22	59
Male	15	41

*Attending school*		
Yes	35	95
No	2	5

*Current school attendance*		
Primary school	11	31
Secondary school	24	69

*Type of caregiver*		
Mother	12	32
Grandmother	9	24
Mother and father	6	16
Aunt	5	15
Father	3	8
Older sibling	2	5

*Orphan status of the adolescents*		
Nonorphan	22	60
Maternal orphan	15	40

**Table 2:** Age of disclosure, self-disclosure, and person who disclosed HIV status  to the adolescent.

	Number	Percent
*Who disclosed HIV status to adolescent *		
Told by mother at home	12	32
Told by nurse at the clinic	9	24
Told by father at home	3	8
Told by grandmother at home	4	11
Told by aunt at home	2	5
Told by counselor at the clinic	3	8
Told by doctor at the clinic	1	3
Knew before but later told by older sister at home	1	3
Overheard nurse telling mother	1	3
Told by stepmother at home	1	3

*Age of disclosure *		
5–9 years	5	14
10–12 years	12	32
13–16 years	20	54

*Duration since disclosure*		
1–4 years	25	67
5–9 years	11	30
10 years	1	3

*Self-disclosure to others (n = 36)*		
Yes	8	22
No	28	78

*Person adolescents self-disclosed their status to (n = 8)*		
Friend/s	4	50
Teacher	4	50

*Duration on ART (n = 36)*		
1–4 years	20	56
5–9 years	9	25
10 years	7	19

*Missed ART dose in past month*		
Yes	7	19
No	30	81
